# Revealing the full extent of households’ experiences of HIV and AIDS in rural South Africa

**DOI:** 10.1016/j.socscimed.2007.05.002

**Published:** 2007-09

**Authors:** Victoria Hosegood, Eleanor Preston-Whyte, Joanna Busza, Sindile Moitse, Ian M. Timaeus

**Affiliations:** a Centre for Population Studies, LSHTM, UK; b Africa Centre for Health and Population Studies, UKZN, Mtubatuba, KwaZulu Natal, South Africa; c HIVAN, Durban, KwaZulu Natal, South Africa; d UNDP, Pretoria, South Africa

**Keywords:** HIV, Households, Impact, Mortality, Morbidity, South Africa

## Abstract

Households experience HIV and AIDS in a complex and changing set of environments. These include health and welfare treatment and support services, HIV-related stigma and discrimination, and individual and household social and economic circumstances. This paper documents the experiences of 12 households directly affected by HIV and AIDS in rural KwaZulu Natal, South Africa, between 2002 and 2004. The households were observed during repeated visits over a period of more than a year by ethnographically trained researchers. Field notes were analysed using thematic content analysis to identify themes and sub-themes. This paper focuses on three dimensions of household experience of HIV and AIDS that have received little attention in HIV and AIDS impact studies. First, that experience of HIV and AIDS is cumulative. In an area where population surveys report HIV prevalence rates of over 20% in adults, many households face multiple episodes of HIV-related illness and AIDS deaths. We describe how these challenges affect perceptions and responses within and outside households. Second, while over 50% of all adult deaths are due to AIDS, households continue to face other causes of illness and death. We show how these other causes compound the impact of AIDS, particularly where the deceased was the main income earner and/or primary carer for young children. Third, HIV-related illness and AIDS deaths of household members are only part of the households’ cumulative experience of HIV and AIDS. Illness and death of non-household members, for example, former partners who are parents of children within the households or relatives who provide financial support, also impact negatively on households. We also discuss how measuring multiple episodes of illness and deaths can be recorded in household surveys in order to improve quantitative assessments of the impact of HIV and AIDS.

## Introduction

Families and households in rural South Africa face the adverse demographic, social and economic consequences of the severe HIV epidemic. In areas of rural KwaZulu Natal where the prevalence of HIV in adults (15–54 years) had reached 23% in 2003/2004 (Welz et al., 2007), households will have experienced multiple HIV-related illness and AIDS deaths of their own members, among their extended families, and in their wider social networks. Empirical studies conducted in South Africa and Kenya have shown that households in which multiple adult deaths have occurred within the previous two years are at statistically higher risk of dissolution, migration, and reduced economic status compared with households that experienced a single or no adult deaths (Hosegood, McGrath, Herbst, & Timæus, 2004; Mutangadura, Mukurazita, & Jackson, 1999; Yamano & Jayne, 2004). Several reviews have noted the limited ability of study designs with short follow-up periods to identify the full array of social and economic effects of ill-health and death (Booysen & Arntz, 2003; Bray, 2002; Heuveline, 2004; Loevinsohn & Gillespie, 2003; Russell, 2004). However, many impact studies in southern Africa restrict their measurement of ill-health and death to that of a single index adult or to episodes within a short period. Unless multiple events within a household over time are explicitly captured, many of the complex interactions between past events and current circumstances in a household, and the way in which multiple events shape a household's social and economic environment and its ability to respond to subsequent deaths, will be obscured.

In no society do households exist in isolation. In South Africa the Apartheid-era control of settlement mainly of the African population and the entrenched migrant labour system has resulted in the phenomenon of “stretched” households (Jones, 1993; Ross, 1996; Russell, 2005; Spiegel, Watson, & Wilkinson, 1996; Spiegel, 1986). In the rural area where this study was conducted, 23% of all household members are resident elsewhere (Hosegood & Timæus, 2005). Thus, strong social and financial ties exist between households living in different places, and the impact of illness and deaths may extend across several residential groups (Hosegood, Benzler, & Solarsh, 2005). Such inter-personal and inter-household relationships are not necessarily supportive and may become marred by conflict, disappointment and suspicion, and weakened by distance, differing priorities and changing circumstances (Niehaus, 1994; Preston-Whyte, 1978; Spiegel, 1980, 1987; Van der Waal, 1996).

To explore the impact of HIV and AIDS on rural households in an area with a very rapid and severe HIV epidemic, we conducted a two and a half year ethnographic study of 20 households in rural South Africa. In this paper we describe the past and current experiences of, and responses to, multiple illness and death in 12 of the study households, which were observed for more than one year. Life histories collected from household respondents included information about events occurring in other households that are linked to the households investigated here through kinship or social ties. We also discuss ways of modifying household surveys in order to improve quantitative assessments of the impact of HIV and AIDS.

## The study area

This study was conducted in the northern KwaZulu Natal, South Africa. The area includes land under the Zulu tribal authority, formerly part of a homeland under the Apartheid-era Bantu Authorities Act of 1951 (Crankshaw, 1997), and an urban area under municipal authority, formerly for Black residents. Infrastructure development and population density across the area are heterogeneous, ranging from fully serviced town houses to isolated rural homesteads without water, electricity or sanitation (Hosegood & Timæus, 2005). The population is highly mobile; approximately 40% of male and 35% of female adult household members (18 years or older) reside outside the area but return periodically and maintain social relationships with households (Hosegood, McGrath et al., 2004). Twenty-eight percent of household heads are non-resident. Although most of the study area is rural, few households are engaged in subsistence agriculture, and most are dependent on waged income and state grants. Rates of unemployment (22% of people aged 15–65 in 2001) are high (Case & Ardington, 2004).

KwaZulu Natal is the province with South Africa's highest HIV prevalence rate among antenatal clinic attendees. A 1998 antenatal survey in the largest clinic within study area found that 41% of pregnant women were HIV infected [95% confidence interval (CI), 34.7–47.9] (Wilkinson, Connolly, & Rotchford, 1999). In 2003/2004, data from population-based HIV surveillance in the area showed that 27% of resident women (15–49 years) and 14% of resident men (15–54 years) were HIV infected (Welz et al., 2007). AIDS is the leading cause of death, accounting for 73% of female and 61% of male deaths (15–44 years) in 2000 (Hosegood, Vanneste, & Timæus, 2004). Between 2000 and 2002, 8% of households experienced the death of a member 15 years and older due to AIDS (Hosegood, Vanneste et al., 2004). In 2004, 7% of children under 18 years were maternal orphans, 13% were paternal orphans and 2% were double orphans (Hosegood, McGrath et al., 2005; Hosegood, et al., 2007).

## Methods

This paper presents an analysis of field notes collected between 2002 and 2004 as part of a study examining the experiences of households directly affected by HIV and AIDS. The study collected ethnographic data through close observation by research staff 20 households caring for at least one adult with disease symptoms indicative of AIDS. Households were identified by volunteers in a local home-based care programme, by research nurses visiting households in the area, nurses or through opportunistic contacts by research assistants in the course of their fieldwork.

Depending on events taking place in the study households, the three research assistants might sometimes spend several days with one particular household or person, while at other times they would visit for an hour or so every couple of weeks for an update. Research assistants were flexible in taking part in household activities: they went with respondents to hospital and welfare services, joined them on visits to neighbours or the market, and attended and helped during funerals. In addition, they accompanied home-based care volunteers on their household visits as well as attended their meetings and training sessions. Some study participants and volunteers actively updated the research assistants themselves, for example, letting them know about hospital admissions. Two of the three research assistants lived in the same community and were in daily contact with many volunteers and household members. All were women and spoke fluent Zulu and English. Two of them had post-graduate-level education; all were trained in ethnographic methods. Moitse, one of this paper's co-authors, had previous ethnographic study experience. Field notes were written up immediately after visits to study households, and were managed and coded using the N6 software (QSR).

The analysis presented in this paper is based on detailed field notes from visits to 12 of the 20 households that were observed for more than 1 year. Information on illness and death of non-household members who were connected to the household was also collected. These field notes were grouped into themes and sub-themes as they emerged in order to explore both content (narrated events, described signs and symptoms, etc.) and discourse (perceived causes, language intimating stigma or support, etc.). Pseudonyms are used throughout this paper.

As no clinical tests were conducted during fieldwork, individuals’ HIV status and a diagnosis of AIDS are based on respondents’ self-reports of having received a positive test result (or report by proxy, after a death), the reports of other household members, volunteers, or on our own interpretation given the signs and symptoms exhibited immediately prior to death. During the period of this study, no antiretroviral treatment programmes were being provided in the area by the government health services or home-based care voluntary organizations.

Prior to starting fieldwork the study was discussed with the Community Advisory Board of the Africa Centre for Health and Population Studies; ethical approval was granted by the Faculty of Medicine Ethics Committee of the University of KwaZulu Natal. The head and other senior members of the household gave verbal consent allowing research assistants to visit and subsequently make notes about their observations and conversations. The research team provided support to the study households in a variety of ways including arranging transport in emergencies and introducing households to home-based care volunteers and the Africa Centre social worker.

## Findings

When we first met the households their daily lives were dominated by efforts to cope with the direct and indirect effects of having a sick member with symptoms indicative of AIDS. In seven households, one or more members died of AIDS during the study. For most households, the episode of illness that triggered our initial visits was only a small part of their cumulative experience of HIV and AIDS. Half of the households had experienced the death from AIDS of another member before they were enrolled in the study. Household responses to HIV-related illness and AIDS deaths were conditioned by other historical and contemporary circumstances and events. These predominantly poor households faced insecurity in many aspects of their lives, and adverse consequences of multiple experiences of HIV and AIDS were often compounded by other events, for example, additional episodes of illness or death due to other causes. In addition, the impact of HIV-related illness and AIDS deaths of closely connected people outside the household had social, emotional and economic repercussions for the households.

### Multiple episodes of HIV-related illness and AIDS deaths within households

#### Case study 1

**Fig. 1:**
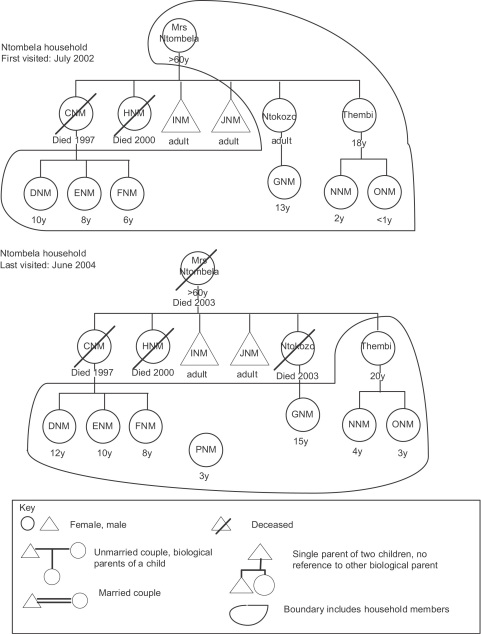
Case study 1, a genogram representing the Ntombela household at the start and the end of the study.

Mrs. Ntombela, a divorced pensioner in her 60s, openly discussed how HIV and AIDS had affected her family. The household circumstances at the time of enrolment and at the end of the study are shown in Fig. 1. When we first met the household in 2002, Mrs. Ntombela had already lost two adult daughters due to AIDS and was living with her two young adult daughters and six grandchildren. Both deceased had been working prior to becoming ill. Three of the grandchildren had come to stay with Mrs. Ntombela after their mother had died. The households’ financial situation was difficult, relying on income from Mrs. Ntombela's government old age pension and a daughter, Ntokozo, who was employed as a domestic worker nearby. For a while her grandchildren were unable to attend school because the school fees could not be paid. This was overcome when Mrs. Ntombela received child support grants for three of the youngest children. Mrs. Ntombela was very concerned about the future. Two of her orphaned grandchildren were sick and Mrs. Ntombela suspected that they were HIV positive. Her oldest surviving daughter, Ntokozo, was also often sick with repeated episodes of T.B., and Mrs. Ntombela feared that Ntokozo was also HIV positive. Eventually Ntokozo had to stop working but fortunately her younger sister, Thembi, was taken on in her place. In February 2003, Ntokozo died of AIDS, leaving her teenage daughter in the care of Mrs. Ntombela. Over the next 6 months, Mrs. Ntombela's own health deteriorated and she was hospitalized twice with T.B-related pneumonia. Thembi stopped working in order to look after her mother, her own children and her four nieces. Mrs. Ntombela died in November 2003. On the day of the funeral, one of Thembi's cousins abandoned a 3-year-old child at the house leaving Thembi as the only adult caring for seven children. Although difficult before, the financial situation deteriorated further as the pension income and child support grants ceased upon Mrs. Ntombela's death. The family managed with help from Mrs. Ntombela's brother and a neighbour until Thembi started to receive government foster care grants for the younger children.

As this case illustrates, households in this area are frequently extended with three generations living together. A common arrangement is for a household headed by older people to also include their unmarried adult children and their grandchildren. Thus, in a population with a generalized HIV epidemic and very high HIV prevalence, extended households may have to respond to the illness and death of more than one adult sibling, their partners, and their young children. Repeated deaths led to increases in the dependency ratio of households. In all households where an adult member had died before the study started, the deceased had been a parent. Thus, households were already caring for one or more orphaned child at the time another adult died. Many of the caregivers were young and were also HIV positive. Thus, some children lost not only one or both parents but their subsequent primary caregiver too.

The economic resources of all households experiencing repeated episodes of illness and death and an increased dependency ratio were severely affected. Increases in expenditure on health care and funerals often came at the same time as losing the income from the patient or their carer and result in deepening financial difficulties. Households in our study often found themselves unable to afford health care, schooling, or adequate food, and several borrowed money. The situation was most acute before and immediately after a death because of the difficulty in obtaining government cash grants for disability and caring for orphans. The delay in obtaining these grants is due in part to the time it takes to complete the application process. As well as because some of the poorest households did not have enough money to apply for a grant, for example to pay for transport to health and welfare offices or in some cases, sadly, to pay the bribes demanded in order to supply documents or process applications. Case studies detailing the experience of the study households in applying for, and receiving grants have been published elsewhere (Moitse & Hosegood, 2003; Zungu, Moitse, & Hosegood, 2003).

There were also psycho-social consequences for households experiencing multiple HIV-related illness and AIDS deaths that evolved more gradually, in particular stigma and discrimination. Over time, with each new episode of illness and death, respondents felt increasingly isolated and stigmatised by relatives and neighbours. When describing how they believed that they were seen by neighbours and service providers, respondents used stigmatizing labels such as ‘*diseased*’ and ‘*poor*’. Several respondents felt strongly that their impoverished circumstances deterred people from visiting or helping them out, and that poverty exacerbated the stigma around HIV and AIDS. A succession of deaths among previous sexual partners was often seen by family and neighbours as a clear sign that the deceased and surviving partners were HIV positive, and was accompanied by considerable discussion about sexual behaviour. Such intense scrutiny of their partnerships and sexual behaviour was distressing to surviving partners, some of whom felt criticized, blamed, and stigmatised. This is illustrated by the experience of Mr. Nsele described in case study 2.

### Stigma connected to HIV and AIDS

#### Case study 2

Mr. and Mrs. Nsele were living with their four children at the start of the study. Mrs. Nsele was already very sick and was being cared for primarily by her husband until he also became ill. Although it is customary for neighbours to visit households affected by illness, the family felt ostracized, with the only visitors being one neighbour, Mr. Nsele's mother and a home-based care volunteer. After his wife died, Mr. Nsele became increasingly conscious that his past sexual behaviour was being talked about and that he was accused of infecting his wife. His perception seemed to be well-founded: home-based care volunteers and neighbours related stories about his past escapades and extra-marital relationships to the study team. They were highly critical, some even suggesting that his poor health was ‘deserved’. As he sickened, Mr. Nsele felt unsupported and stigmatized because of his wife's death, his poverty and his own ill-health. He responded by shunning most offers of help and relying increasingly on his teenage sons. He was less welcoming of visits by the research team. After his death, his own relatives became suspicious that his wife's family had caused his death through bewitchment because of the ‘*evil things’* he had done to their daughter. Tensions between the families were somewhat eased by his forthright mother, who stated publicly at his funeral that nobody had killed her son rather that he had died from AIDS.

While perceived and enacted stigmatizing behaviour towards individuals and households affected by HIV and AIDS was common, pre-existing tensions and conflicts with relatives and neighbours influenced the form that such behaviour took. Many people who disclosed that they, or the person they cared for, were HIV positive, believed that they had been infected because of witchcraft by someone outside the family. Often poor relationships between ex-partners or the family of ex- and deceased partners deteriorated further as families placed the ‘blame’ for HIV infection on one another. Feeling bewitched added to the sense of helplessness and victimization expressed by many households who had experienced multiple deaths in quick succession.

### Experiences of HIV and AIDS compounded by other causes of illness and death

While the HIV epidemic has resulted in a dramatic rise in adult mortality over the last two decades in the study area, nearly half of all adult deaths are due to causes other than AIDS (Hosegood, Vanneste et al., 2004). Consequently, in several study households the burden of HIV and AIDS was compounded by illness and death due to other causes including tuberculosis, strokes, violence and accidents.

#### Case study 3

The Msweli household was repeatedly hit by illness, death and disability before and during the study. At the start of the study, the household consisted of Rose, a widowed pensioner, her adult daughter Zodwa, and five grandchildren, none of whose parents were members of the household. Three children were orphans. Two of Rose's adult children had died. One daughter had died of AIDS leaving Jacob who was 8 years old. Jacob was also HIV positive and suffered from repeated bouts of tuberculosis and eye infections. Zodwa, his aunt, was Jacob's primary caregiver. Jacob's father lived elsewhere; he was sick and unable to send money for Jacob. Zodwa and Jacob were very attached to each other. Zodwa spent a lot of time and money taking Jacob to see different doctors. She was supported in doing this by her boyfriend and her mother who helped pay for some of the health care and transport costs. During the study Zodwa too was diagnosed as HIV positive and started to become ill.

The toll that HIV and AIDS took on this household was compounded by illness and death due to other causes. Two of the grandchildren in this household were 12-year-old twins, abandoned a few years before by their mother and taken in by Rose. One twin had severe mental and physical disabilities and required constant care, the other had moderate learning and behavioural difficulties. Zodwa had been injured 2 years before in a car accident. During the study, Rose had a stroke and was hospitalized for several weeks. The family felt that Rose's stroke had been brought about by the stress of repeated traumas in the family. Shortly afterwards, Phumzile, another married daughter of Rose's moved into the household. Phumzile had previously been living in Johannesburg but had been ill for some time with haemophilia. She moved with her young sons to live with her mother after her husband refused to care for her anymore. She died within a month of arriving due to complications. The role of caring for the numerous sick and dependent children and adults moved back and forth between Rose and Zodwa as they struggled with own health problems.

Non-AIDS disability, illness or death can have the same or a greater economic and social impact than AIDS depending on the person affected. Illness and deaths of older people were common in many households and, given their pivotal role in the households they belonged to, their deaths resulted in major changes for households. Five of the households studied were headed by someone over 50 years. All the older women in the study were the primary caregiver of at least one grandchild; in some cases because the children had been orphaned but in others because their parents were absent from the household for employment-related or other reasons. In addition to their role as caregivers, several households were financially dependent on an older person's government pension.

Many household respondents and volunteer caregivers described their distress with having to respond to seemingly relentless bouts of severe illness and repeated bereavements. Past experiences primed carers to expect that death was inevitable. Some respondents focused their expressions of fear about the future on worries about financial and practical problems. Others dwelt on the reasons why they were being repeatedly affected by illness and death. Mthokozi, a 5-year-old whose mother had died of AIDS a year before, reflected the general mood in the Msweli household when he talked about his sick aunt: “My mother was sick like Zodwa and I think that Zodwa will die because her sickness is the same as for my mother.” Another adult respondent added: “What have we done wrong in this household? Months back Phumzile died and now Zodwa is sick.”

### Multiple HIV-related illness and AIDS deaths of people linked to the households studied

The impact of HIV and AIDS was not restricted to experiences within the household. HIV-related illness and AIDS deaths of people who lived elsewhere, but were connected to the households studied, also had repercussions on study households. When discussing the household's experience of HIV and AIDS respondents often explained a situation by describing events, so to speak, ‘off-stage’.

#### Case study 4

**Fig. 2:**
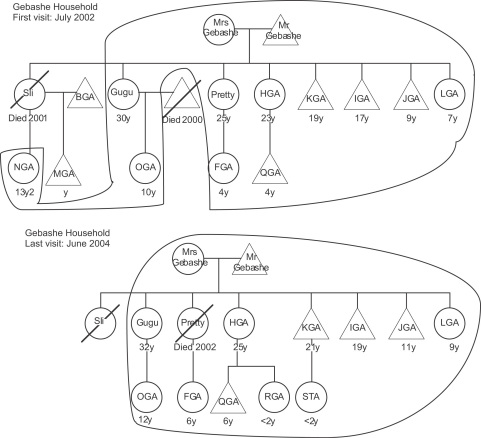
Case study 4, a genogram representing the Gebashe household at the start and the end of the study.

The Gebashe household was large. Headed by Mr. Gebashe, it included his wife, seven children and five grandchildren. The household composition at the start and end of the study are shown in Fig. 2. At the start of the study, the household's main source of income was a government disability grant that Mr. Gebashe received after losing his mining job following a stroke. The Gebashe's eldest daughter, Delisiwe, died of AIDS in 2001. Prior to her death, Delisiwe, who had been living in a separate household with her husband and child, had a relatively well-paid job and provided financial support to her parents. Delisiwe's oldest child was fostered by her maternal relatives while the youngest continued to live with his father. Another daughter, Gugu had also been living separately with her husband and child but, in 2000, Gugu's husband died of AIDS. Gugu had been working as a domestic worker but when she became ill, she stopped working and she and her child moved back to live with her parents. During the study, Gugu disclosed that she was HIV positive and had tuberculosis. A third daughter, Pretty, who was living and working elsewhere became sick and returned home, accompanied by her child. After a long period of illness, which included T.B, shingles and respiratory problems, she was diagnosed HIV positive and died in 2002. Her daughter remained with the Gebashes. During the study two further grandchildren were born and became part of the household.

Illnesses or deaths occurring in one household can result in people moving to another household motivated by the need for care or financial assistance, thus changing the composition and dependency ratio of both households. At the start of the study, almost all the households included children whose parents were not married or co-habiting. Although in most cases little or no financial and material support was given by former partners or parents outside the household, such assistance as was given typically ceased when the person became ill or died because the deceased person's relatives were unwilling to continue making maintenance payments. Therefore, this support stopped at the same time that the affected household was undergoing extreme financial difficulties due to income loss, increased costs, and while waiting to receive government grants. This not only exacerbated household poverty but also fostered respondents’ feelings that they were unsupported and stigmatized, and heightened pre-existing conflicts between households.

## Discussion

The households in this study experienced repeated episodes of illness and death during two and a half years of observation. These shocks were often closely followed by or occurred at the same time as other experiences of HIV and AIDS within the household or in inter-connected households. The age-specific patterns of sexual and vertical transmission of HIV, together with the social context in rural South Africa, in which households are commonly extended to include unmarried, adult children, create a situation in which multiple HIV-related illness and AIDS deaths are likely to occur. Such repeated episodes pushed these generally poor households into deepening financial crisis. The increased expenditure due to illness and death leading to food insecurity and problems with keeping children in school have also been reported by other studies conducted in Africa (Booysen, 2002; Guiness & Alban, 2000; Patel, 1995; Yamano & Jayne, 2004). The deaths of more than one young adult parent increased the dependency ratios in many households. In some cases, foster parents were themselves HIV positive.

Multiple experiences of HIV-related illness and AIDS deaths, compounded by other causes of death, elicited strong emotional responses from surviving household members. Distress about the apparently relentless way in which their families were being struck by illness and death added to their fear of HIV and AIDS stigma, exacerbating their feelings of isolation and lack of support from relatives and neighbours. The study team observed that neighbours and home-based care volunteers considered multiple deaths of young people to be ‘proof’ that a household had members who were HIV positive. Neighbours who had previously made visits to see young people when they were ill, reported becoming concerned about HIV when other members of the household also fell ill and consciously ceasing to visit.

Our observations suggest that while AIDS is an important cause of ill-health and mortality in young adults and children, illness and death due to other causes compound the negative consequences of the HIV epidemic. In some cases, the effect of these other deaths on the household was critical, particularly when the person who died was a pivotal member of the household, such as a senior member of the family or an older pensioner. The qualitative data provide contextual information in addition to findings from an quantitative study of longitudinal, population-based data in the same area that showed that multiple adult deaths, independent of cause, are significantly predictive of household dissolution and migration (Hosegood, McGrath et al., 2004). The robust response of households that experience a single death is exhausted by having to respond with the demands placed on them if a second or third death follows in quick succession. This was mirrored by diminishing support from relatives and neighbours either unable or unwilling to support increasingly impoverished households. Multiple deaths exhausted all financial reserves in several study households. With no income or assistance coming into the household, they entered a very bleak period unable to buy sufficient food or pay for school fees. All households reaching this extreme situation during the study eventually received financial assistance through government foster care or child support grants. Thus, applying the phrase ‘coping with HIV and AIDS’ to many of the study households would have been misleading. As noted by Loevinsohn and Gillespie (2003), many distressed households cannot be considered to be ‘coping’ with the impact of HIV and AIDS since the word implies a degree of self-sufficiency, that responses made by the household are neither too costly or irreversible, and it does not account for the long-term effects.

The study has also highlighted that households are not isolated from events occurring in their immediate social networks as well as elsewhere in the community. Adult deaths in one household may have repercussions for several other households connected to this household. The results of this qualitative study have implications for the design and interpretation of studies designed to quantify the impact of AIDS deaths on households. As suggested by Booysen and Arntz (2003) in their review of impact studies, the extent of the impact of HIV and AIDS on affected people and households only becomes manifest when households are observed for longer periods. However, this is only part of the methodological challenge. Our findings suggest that longitudinal household studies risk attributing the cumulative impact of several deaths to the death that occurred most recently when they do not record a more complete history of household experiences of illness and death. Understanding the historical antecedents of contemporary social and economic circumstances allows for a more realistic assessment of the burden of illness and death faced by households. Extreme social and welfare outcomes, for example household dissolution, or children being taken out of school or sent to live with other households, may be the final culmination of a series of AIDS and non-AIDS episodes of illness and death occurring within and outside the household.

Our finding that a household's experience of HIV and AIDS is only partially revealed without information about events outside the household is probably the most difficult to address in population-based surveys and other questionnaire-based studies (Save the Children, 2004). Such information is difficult to obtain through exclusively household-focused approaches where respondents are asked to provide structured information about recent episodes of illness and mortality of people listed as household members. Modifying data collection to capture events that occur *off-stage* presents substantial challenges for systematic recording and introduces complexities in data management and analysis. Thus, a recent paper has explicitly called for greater use of case studies as an approach that captures household experiences and responses more holistically (Russell, 2004). However, more can be done to ensure that larger household surveys capture information about ‘stretched household’ arrangements and relationships with people outside the household. For example, information can be collected about non-resident household members, about whether a child's mother and father is alive or has died, about financial support from such individuals, and about whether a person's last relationship ended because of the death of their partner. Without a more nuanced understanding of how households respond to the high burden of morbidity and mortality, the data collected by community social and health programmes may fail to identify some of the most vulnerable households and individuals.
